# Anti-tumor activity of fenretinide complexed with human serum albumin in lung cancer xenograft mouse model

**DOI:** 10.18632/oncotarget.2038

**Published:** 2014-05-28

**Authors:** Sandra Durante, Isabella Orienti, Gabriella Teti, Viviana Salvatore, Stefano Focaroli, Anna Tesei, Sara Pignatta, Mirella Falconi

**Affiliations:** ^1^ DIBINEM—Department of Biomedical and Neuromotor Sciences, University of Bologna, Via Irnerio 48, 40126, Bologna, Italy; ^2^ FaBiT–Department of Pharmacy and Biotechnology, University of Bologna, Via San Donato 19, 240127, Bologna, Italy; ^3^ Biosciences Laboratory, Istituto Scientifico Romagnolo per lo Studio e la Cura dei Tumori(IRST) IRCCS, Biosciences Laboratory, via P. Maroncelli 40, 47014, Meldola, FC, Italy

**Keywords:** Fenretinide, caveolin-1, ACSVL3, Albumin, lung cancer

## Abstract

Sufficient knowledge regarding cellular and molecular basis of lung cancer progression and metastasis would help in the development of novel and effective strategies for the treatment of lung cancer. 4HPR is a synthetic retinoid with potential anti-tumor activity but is still limited because of its poor bioavailability. The use of albumin as a complexing agent for a hydrophobic drug is expected to improve the water solubility and consequently their bioavailability.This study investigated the antitumor activity of a novel complex between albumin and 4-HPR in a mouse model of human lung cancer and focuses on role and mechanism of *Cav-1* mainly involved in regulating cancer and *Acsvl3* mainly connected with tumor growth.

Their expressions were assayed by immunohistochemistry and qRT-PCR, to demonstrate the reduction of the tumor growth following the drug treatment. Our results showed a high antitumor activity of 4HPR-HSA by reduction of the volume of tumor mass and the presence of a high level of apoptotic cell by TUNEL assay. The downregulation of *Cav-1* and *Acsvl3* suggested a reduction of tumor growth.

In conclusion, we demonstrated the great potential of 4HPR-HSA in the treatment of lung cancer. More data about the mechanism of drug delivery the 4HPR-HSA are necessary.

## INTRODUCTION

Lung cancer has become the most common malignant tumor and the major cause of cancer deaths worldwide. The overall 5-years survival rate for lung cancer is only 15% [1-2]. Although combination chemotherapy has improved the prognosis of patients with non small cell lung cancer (NSCLC), there are still many patients who have initial resistance to chemotherapy or develop drug resistance after several courses of chemotherapy [3]. The development of new therapeutic drugs for the treatment of NSCLC has become an important area for research in cancer therapy.

Caveolins, 21 to 25 kDa integral membrane proteins, are the principal structural proteins in caveolae. Caveolin-1 (Cav-1) binds to signaling molecules such as G-proteins, Src family tyrosine kinases, receptor tyrosine kinases (epidermal growth factor receptor), protein kinase C and eNOS and can functionally inactivate the enzymatic activity of these molecules [4]. The gene encoding human caveolin-1 is located in on chromosome 7q31.1, downstream of the D7S522 locus, in the fragile site FRA7G [5, 6]. Deletions of this region are frequently noted in human cancers including squamous cell carcinoma [7], sarcoma [8], prostate carcinoma [9], renal cell carcinoma [10] and ovarian carcinoma [11]. Furthermore lower caveolin-1 expressions were reported in some human malignant tumors [12-14]. These results suggested that caveolin-1 might be involved in oncogenesis as a tumor suppressor [6]. In contrast caveolin-1 overexpression was also reported in some human carcinomas [15]. In addition it was reported that caveolin-1 expression in tumor cells affects the clinical outcome and prognosis of patients with squamous cell carcinoma of the lung [16]. That suggests caveolin-1expression is significantly correlated with an advanced pathological stage and a poor prognosis in non-small cell carcinomas of the lung (NSCLC) [16]. Recent studies have demonstrated that Cav-1 can directly confer the anoikis resistance in NSCLC by the interaction with its antiapoptotic partner Mcl-1 protein and prevent the latter protein from the degradation by the ubiquitin-proteasomal system [17]. These results suggest that caveolin-1 plays a biological role in the regulation of tumor invasiveness, and that it should be regarded as a prognostic marker for these tumors and as a possible target for new drugs [8].

At a physiologic level caveolin functions as a scaffolding protein to organize and concentrate specific lipids and lipid-modified signaling molecules in particular cell membrane microdomains inducing caveolae formation [18]. The main role of caveolae is the uptake and transport of macromolecules from the extracellular space to the intracellular environment.

Albumin is one of the caveolar ligands. Indeed, it undergoes continuous receptor-mediated transcytosis across endothelia via caveolae [19,20]. Being albumin one of the most abundant serum proteins that functions as a carrier for fatty acids, steroids and thyroid hormones, the caveolae-mediated transcytosis of albumin represents the mechanism by which these important molecules are distributed from the vascular space to surrounding tissues [21,22]. Very Long-Chain Acyl-CoA Synthetase 3 (Acsvl3) catalyzes the ATP-dependent thioesterification of fatty acids (FA) to coenzyme A (CoA). This “activation step” is necessary for FA to participate in nearly all subsequent metabolic reactions. In addition to their metabolic functions, these enzymes were also proven to be FA transport proteins (FATP). Recent studies showed that upregulation of Acsvl3 is correlated with poor prognosis in lung cancer because it supports malignancy by altering tumor cell metabolism [23].

Fenretinide or N-4-hydroxyphenyl-retinamide (4-HPR) is a synthetic retinoid which emerged as a promising anticancer agent based on numerous in vitro and animal studies as well as chemoprevention clinical trials [24-37]. Despite its excellent tolerability the therapeutic efficacy of fenretinide is still limited because of its poor bioavailability. The strong hydrophobic character of fenretinide limits its solubility in blood and biological body fluids thus limiting its bioavailability towards tumor cells and consequently its therapeutic activity.

Fenretinide encapsulation in amphiphilic micelles [38-41] or PLA microspheres [39] has proven increased bioavailability and antitumor activity.

In this study we complexed fenretinide with Human Serum Albumin [HSA] with the aim to enhance its bioavailability through an improvement of its aqueous solubility and to exploit the Albumin affinity to cav-1 to favour the uptake of the complex in the tumor cells expressing high levels of cav-1.

We evaluated the antitumor activity of the complex in a mouse xenograft model of human lung adenocarcinoma. Apoptotic cell death was detected by ematossilin and eosin staining and TUNEL assay. Cav – 1 and ACSVL3 expressions were chosen as tumor biomarkers to demonstrate the reduction of the tumor growth following the drug treatment.

## RESULTS

### Volume of xenograft tumors after 4HPR-HSA treatment

**Figure 1:**
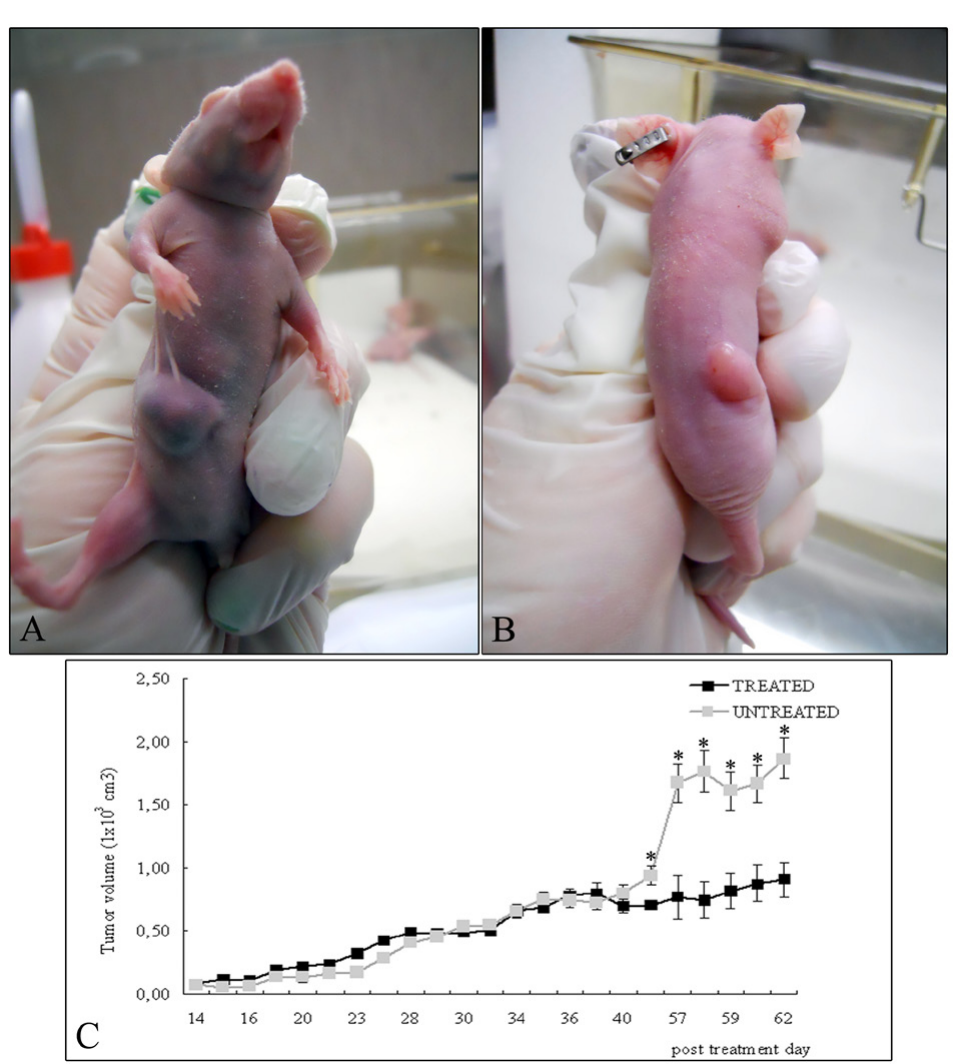
A549 cells were injected into the right flanks of nude mice and tumor size was serially measured until post treatment day 62 (A) Representative photographs of xenograft tumors in situ were taken demonstrating increased A549 tumor size compared with the (B) treated tumors. (C) Over the time tumor volumes showed significant growth retardation in the treated group compared with untreated group (mean ± standard error of the mean; n=10). * p < 0.05 compared to untreated tumors.

Figure 1 shows the xenograft tumors of A549 in the right flank of nude mice not treated (Figure 1A) and after treatment with 4HPR-HSA (Figure 1B) at the end of the experiment. Tumor proliferation curve showed that in the treated mice the tumors grew significantly slower than untreated ones (Figure 1C). HPLC analysis indicated that fenretinide was absorbed in the tumors after administration of the complex indeed a mean drug concentration of 5.7 ± 1.34 uM was obtained at the end of the experiment.

### Hematoxylin and eosin (H&E) staining

**Figure 2:**
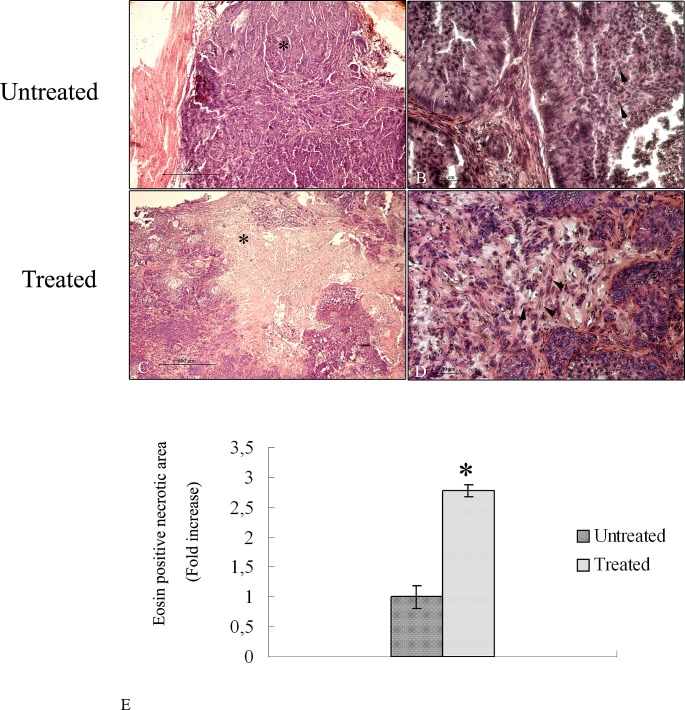
Hematoxylin and eosin stained histopathalogical sections of 4HPR-HSA untreated and treated tumors (A) Low magnification of tumor tissue treated with PBS. A large area of tumor cells is detectable (*) (bar: 600 um). (B) High magnification of tumor tissues treated with PBS. Cells with large nuclei and spherical or spindle shape were observed (arrowhead) (bar: 100 nm). (C) low magnification of 4HPR-HAS treated tumors. A large stained area of dead cells is detectable (*) (bar: 600 um). (C) High magnification of eosin stained area in which several dead cells are observed (arrowhead) (bar: 100 nm). All the results were repeated at least three times. (E) Quantitative analysis of eosin stained area in treated tumors expressed as relative amount compared to eosin necrotic area of untreated tumors (± SD) were assessed by direct visual counting of three fields for each of five slides per each sample at ×10 magnification by Image–ProPlus software. Data are the mean ± SD of three different consistent experiments.

To evaluate the anti-tumor efficacy after treatment with albumin-fenretinide complex, tumors were dissected from mice for histopathalogical analysis. H&E results show that tumor cells with large nuclei of spherical or spindle shape were observed in the tumor tissue treated with PBS (untreated samples) (Figure 2 A-B) while various degrees of dead cells were observed in the group treated with the drug (Figure 2 C-D). Quantitative analysis shows that the necrotic area corresponding to the eosin stained area, is almost three fold larger compared to untreated tumors (Figure 2E).

### Tunel Assay

**Figure 3:**
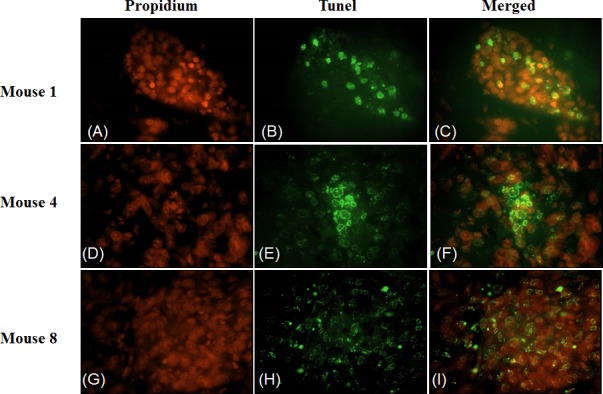
TUNEL assay apoptosis of untreated tumor tissue The images are representative for sections obtained from three different mice of the untreated group. (A) (D) (G) Propidium stained nuclei. (B) (E) (H) TUNEL positive cells representative for apoptotic cells. (C) (F) (I) Merge images of the first and second column. All the images are 600X. All the results were repeated at least three times.

**Figure 4:**
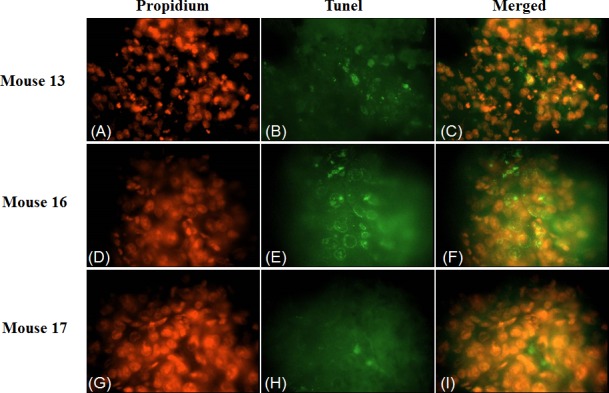
TUNEL assay apoptosis of 4HPR-HSA tumor tissue The images are representative for sections obtained from three different mouse of the 4HPR-HAS treated group. (A) (D) (G) Propidium stained nuclei. (B) (E) (H) TUNEL positive cells representative for apoptotic cells. (C) (F) (I) Merge images of the first and second column. All the images are 600X. All the results were repeated at least three times.

**Figure 5:**
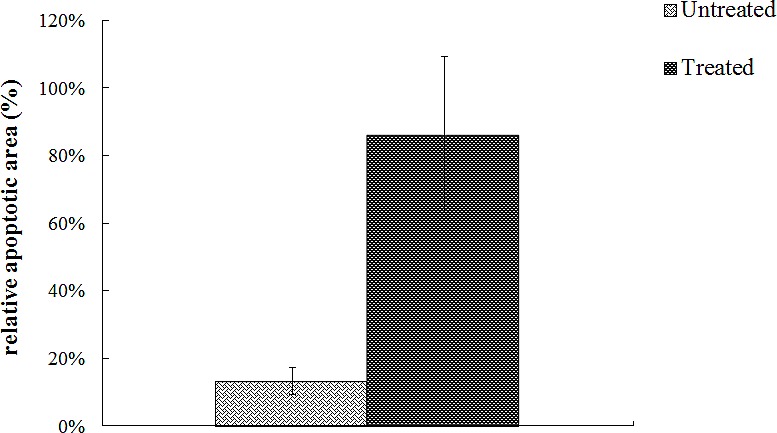
Quantitative analysis of TUNEL positive area expressed as relative amount of treated samples (± SD) compared to untreated ones

To investigate the effect of 4HPR-HSA in the tumor tissue we performed Tunnel assay to demonstrate the presence of apoptotic cells after the drug treatment. We found the drug significantly increases the number of Tunnel-positive cells in the treated group (Fig. 3) compared to the non treated group (Fig. 4) with about 86% apoptotic area when compared with the untreated group (Fig. 5). These results suggest that 4HPR-HSA induces cell death by apoptosis.

### Immunohistochemistry for caveolin – 1 protein

**Figure 6:**
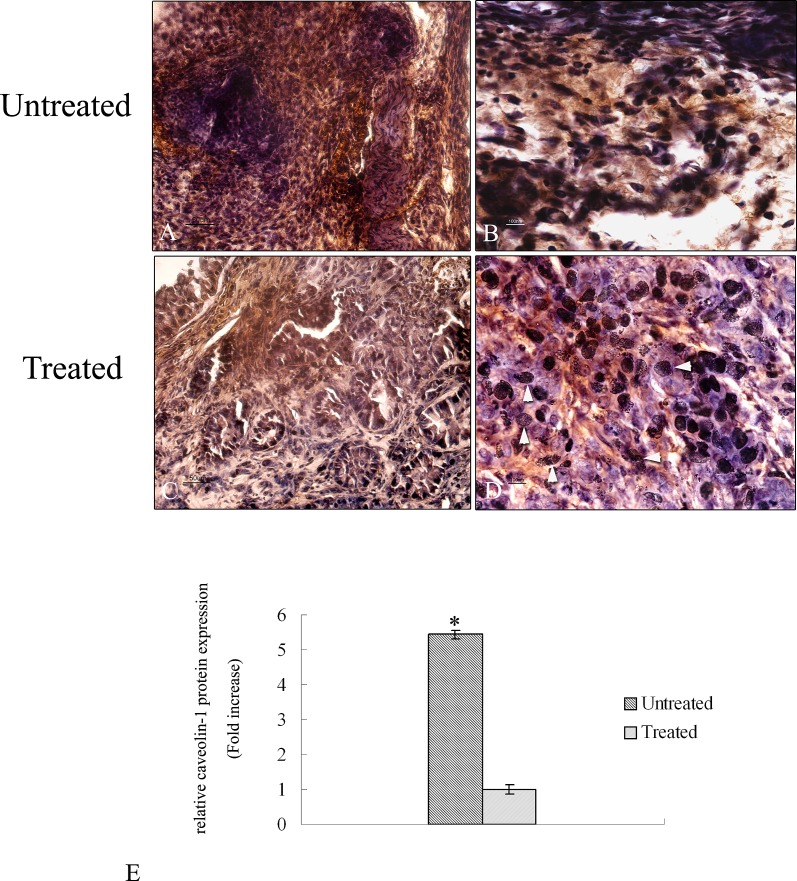
Immunohistochemical analysis of caveolin −1 protein in tumor sections A-D Paraffin – embedded tissues sections were stained with caveolin – 1 antibody and counterstained with hematoxylin. (A) Low magnification (20X) of tumor sections treated with PBS. A high signal corresponding to caveolin – 1 protein is detectable (bar: 50um). (B) High magnification (60X) of tumor sections treated with PBS. Caveolin – 1 signal is mainly localized in the cytoplasm of the cells (bar: 100 nm). (C) Low magnification (20X) of tumor sections treated with 4HPR-HSA. A low signal corresponding to caveolin – 1 protein is detectable (bar: 50um). (D) High magnification (60X) of tumor sections treated with 4HPR-HAS. A few cells showed a cytoplasmic signal corresponding to caveolin – 1 protein. Several damage nuclei were detected (arrowhead) (bar: 100 nm). All the results were repeated at least three times. Quantitative analysis of caveolin-1-positive area expressed as relative amount of treated samples (± SD) compared to untreated ones.

To evaluate the expression of caveolin-1 protein an immunohistochemical analysis was performed in tumor tissue of treated and untreated mice. Figure 6A shows a wide expression of caveolin – 1 protein in untreated samples almost 6 fold higher compared to treated ones (Figure 6E). The staining of the protein is distributed throughout the tumor area in all the analyzed untreated sections. At higher magnification it is possible to observe a high signal of caveolin-1 protein in the cytoplasm of the cells (Figure 6B) in agreement with a poor prognosis of lung tumor. Treated tumor shows a low signal of caveolin-1 protein due to 4HPR-HSA (Figure 6C-D). At higher magnification it is possible to detect several damage of nuclei due to the treatment (Figure 6D).

### Immunohistochemistry for acsvl3 protein

**Figure 7:**
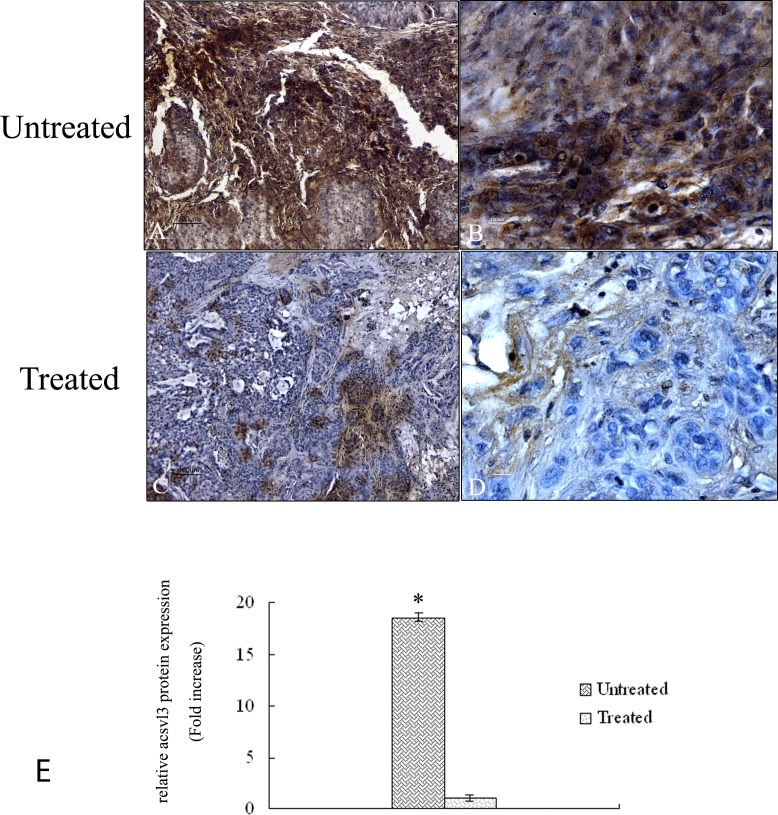
Immunohistochemical analysis of acsvl3 protein in tumor sections A-D Paraffin – embedded tissues sections were stained with acsvl3 antibody and counterstained with hematoxylin. (A) Low magnification (10X) of untreated tumor sections. A high signal corresponding to acsvl3 protein is detectable (bar: 100um). (B) High magnification (60X) of untreated tumour sections (bar: 100 nm). (C) Low magnification (10X) of tumor sections treated with 4HPR-HSA. A low signal corresponding to acsvl3 protein is detectable (bar: 100um). (D) High magnification (60X) of tumor sections treated with 4HPR-HAS. A few cells showed acsvl3 protein staining (bar: 100 nm). All the results were repeated at least three times. Quantitative analysis of acsvl3-positive area expressed as relative amount of treated samples (± SD) compared to untreated ones.

Figure 7 shows the expression of acsvl3 protein in tumor tissue of untreated and treated mice. Untreated samples demonstrated a high signal of the protein throughout the tumor area in all the analyzed tissues (Figure 7A – B). Quantitative analysis demonstrates a protein signal 18 fold higher compared to treated samples (Figure 7E) in agreement with a poor prognosis of lung tumor tissues. Tumor samples exposed to 4HPR-HSA drug show a strong reduction of acsvl3 protein expression (Figure 7C-D) due to the cell death induced by the drug treatment.

### Quantitative Real Time Polymerase Chain Reaction (qRT-PCR)

**Figure 8:**
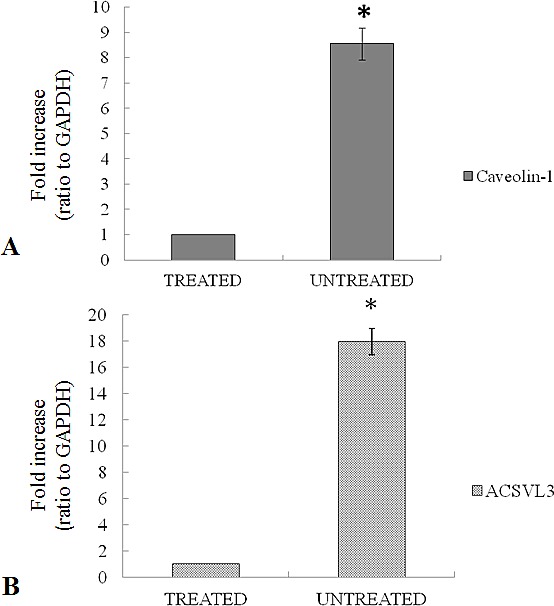
(A) Expression of caveolin-1 mRNA in untreated tumors compared to treated tumors. (B) Expression of acsvl3 mRNA in untreated tumors compared to treated tumors. Each individual assay was performed in triplicates and expressed as mean ± SD; * represents a significant difference of untreated tumors compared to treated tumors, p< 0.05.

The mRNA expression of caveolin-1 and acsvl3 is evaluated by qRT-PCR in untreated tumor tissue and the results are compared to treated samples (Figure 8A-B). Results show a 9 fold up regulation of caveolin-1 in untreated tumor samples compared to samples treated with 4HPR-HSA (Figure 8A) while the mRNA expression of acsvl3 is 20 fold upregulated in untreated samples compared to treated tumor tissues (Figure 8B). These results are in agreement with immunohystochemistry data and demonstrate that the treatment with 4HPR-HSA due to a downregulation of caveolin-1 and acsvl3 mRNA reduces tumor cell proliferation.

## DISCUSSION

Lung cancer has long been recognized as a multi-step process which involves not only genetic changes conferring growth advantage but also factors which disrupt regulation of growth and differentiation. Although combination chemotherapy has improved the prognosis of lung cancer, there are still many patients who have initial resistance to chemotherapy or develop it after several cycles of therapy. Therefore the identification of new therapeutic approaches is extremely important to improve the prognosis of cancer lung patients. To this end the caveolae-dependent transcytosis of albumin in the pulmonary endothelium, triggered by the interaction of albumin with caveolin-1, is emerging as an important tool in the design of novel therapeutic systems based on the use of albumin as a carrier for antitumor drugs [42].

Cav-1 plays an important role in regulating the behaviour of cancer cells including anoikis resistance in NSCLC by the interaction with its antiapoptotic partner [17]. Although combination chemotherapy has improved the prognosis of lung cancer, still many patients have initial resistance to chemotherapy or develop it after several cycles of therapy. The identification of new therapeutic drugs for lung cancer is extremely important to improve the prognosis of lung cancer patients.

Albumin is already used as a drug carrier in several anticancer drug formulations evaluated clinically, including the methotrexate-albumin conjugate (MTX-HSA) [43,44], albumin –binding prodrug of doxorubicin (DOXO-EMCH) [45,46] and albumin paclitaxel nanoparticle (Abraxane). The latter also approved by FDA for treating metastatic breast cancer [47-49].

Fenretinide or N-4-hydroxyphenyl-retinamide (4HPR) is a synthetic retinoid which emerged as a promising anticancer agent based on numerous in vitro and animal studies as well as chemoprevention clinical trials [24-37]. The interest in the clinical use of fenretinide arises from its strong antitumor activity combined with a low toxicity profile. The anticancer activity of fenretinide results from its ability to induce apoptosis in tumor cells by improving diverse signaling molecules including reactive oxygen species, ceramide and ganglioside GD3. In particular fenretinide has been shown to possess cytotoxic activity on a wide variety of experimental models belonging to different types of cancer, including lung cancer [50-53].

Despite its excellent tolerability the therapeutic efficacy of fenretinide is still limited because of its poor bioavailability. Indeed, the strong hydrophobic character of fenretinide limits its solubility in blood and biological body fluids thus limiting its bioavailability towards the tumor cells and consequently its therapeutic activity. Among the different approaches carried out to raise the bioavailability of fenretinide [38-41] complexation with amphiphilic macromolecules provided the best results. In this study we chose Human Serum Albumin as a macromolar complexing agent for fenretinide with the aim to exploit the concomitant ability of albumin to complex fenretinide increasing its bioavailability and to link caveolin-1 providing a targeted drug release towards the populations of cancer cells.

The use of albumin as a complexing agent for fenretinide is believed to improve its water solubility and consequently its bioavailability. Moreover the affinity of albumin for caveolin-1 is believed to target the drug specifically to cancer cell populations expressing high levels of this surface protein. The concomitant improvement in bioavailability and drug targeting should provide a significant increase in the antitumor activity. As reported in the scientific literature lung tumor cells express high levels of caveoli-1 [16].

To evaluate the antitumor activity of 4HPR-HSA A549 human cells of lung adenocaricinoma were implanted subcutaneously on nude mice. All experimental procedures involving animals were performed in compliance with the European Council Directive 86/609/EEC on the care and use of laboratory animals and approved by ethical committed of the University of Bologna (Prot.n. 43-IX/9 of 11/20/2012).

The measurements of the subcutaneous tumor mass reveals a significant reduction of the volume in treated mice compared to control mice suggesting a strong antitumor activity of 4HPR-HSA.

To evaluate the presence of cell death in tumor mass exposed to albumin-fenretinide complex tumors were dissected from mice and processed for histopathalogical analysis. H&E staining showed necrotic areas in the treated tumors three fold larger compared to untreated tumor mass suggesting a high level of cell death induced by the drug.

To better evaluate the presence of apoptosis in the dead areas TUNEL assay was carried out in sections of tumor mass obtained from treated and control samples. Results showed the presence of several apoptotic cells in the treated sample compared to the untreated sample suggesting that 4HPR-HSA induced cell death mainly by apoptosis in agreement with previous data demonstrating the apoptotic effect of 4-HPR [50-53].

Thus based on these previous results an immunohistochemistry analysis was performed on sections of the tumor to evaluate the expression of cav-1 and Acsvl3, NSCLC biomarkers whom overexpression is correlated with tumor growth, invasiveness and metastatic potential [54]. Immunohistochemistry results showed a strong downregulation of caveolin-1 and Acsvl3 proteins in treated mice compared to untreated ones. These data are confimed by qRT-PCR in which a 9 fold upregulation of caveolin-1 in untreated tumor samples and a 20 fold upregulation of acsvl3 in untreated samples were shown.

The Acsvl3 protein is strictly connected with the lipid metabolism and in lung tumor cells its overexpression is responsible for tumor growth [23]. We suppose that the downregulation observed in tumor mass after 4HPR-HSA treatment is responsible of a deregulation of lipid metabolism which reduces tumor growth.

In conclusion our results show a high antitumor activity of 4HPR-HSA demonstrated by the reduction of the volume of tumor mass and the presence of a high level of apoptotic cell death. The downregulation of the tumor biomarkers caveolin-1 and Acsvl3 suggests a reduction of tumor growth. 4HPR-HSA has a great potential in the treatment of lung cancer. More data about the mechanism of interaction between 4HPR-HSA and caveolin-1 protein are necessary. Experiments regarding this interaction and the delivery mechanism of the drug due as a complex with albumin proteins are in progress in our laboratory.

## MATERIALS AND METHODS

### Cell line

The lung adenocarcinoma cell line A549 was purchased by the American Type Culture Collection (ATCC). Cell lines cultured in Dulbecco’s Modified Eagle Medium / F12 (DMEM/F12) (Gibco, Life Technologies, Monza, Italy) supplemented with 10% Fetal Bovine Serum (FBS, Gibco, Life Technologies, Monza, Italy) and 1% penicillin/streptomycin and were then incubated at 37°C in a humidified atmosphere of 5% CO_2_. The cells were subcultured once a week using 1% trypsin (Gibco), expanded in new T75 flasks and maintained at 37°C in a humidified atmosphere of 5% CO2. Cells from passages 2 to 5 were utilized for the experiments described.

### Preparation of albumin-fenretinide complex

The albumin-fenretinide complex was prepared by mixing a fenretinide solution in ethanol with an HSA solution in water. The fenretinide solution obtained by dissolving 200 mg of the drug in 1 ml ethanol, was added to an HSA solution prepared by 1g of HSA in 10 ml water. After stirring 10 min at RT in the dark the mixture was sonicated in a Vibra-Cell VCX 400 Sonicator at 40 kHz for 2 h. The temperature was maintained constant at 40° C by a thermostated circulating water bath. After sonication the mixture was diluted with 40 ml phosphate buffer pH 7.4 and filtered through a 0.22 um filter to obtain an homogeneous suspension of the albumin-fenretinide complex with a mean diameter below 0.22 μm. The fenretinide concentration was determined by a spectrophotometric analysis of the filtrate at 360 nm against a blank consisting of a dispersion of void albumin prepared by the same method.

### *In Vivo* Experiment

Athymic (nu/nu) female nude mice were supplied by Charles River Laboratories and were allowed unrestricted access to sterile food and water. All experimental procedures involving animals were performed in compliance with the European Council Directive 86/609/EEC on the care and use of laboratory animals and approved by ethical committed of the University of Bologna (Prot.n. 43-IX/9 of 11/20/2012). Each experiment employed the minimum number of mice needed to obtain statistically meaningful results. To evaluate the activity of 4HPR-HSA in a mice model A459 cells (5 × 10^6^ cells/mouse in a 200 ul volume of serum-free medium) were implanted subcutaneously in the right flank. Animals were routinely monitored and at the appearance of a visible subcutaneous tumor mass and tumor dimensions were measured every 2 days in two perpendicular directions using calipers. The mice were then randomized into 2 groups of 10 animals.

The animals were routinely monitored and upon the appearance of a visible subcutaneous tumour mass, the dimensions were measured every 2 days in two perpendicular directions using calipers. Tumour volume (mm3) was defined as follows: (W12 × W2) × (π/6), where W1 and W2 are the largest and smallest tumour diameters (mm), respectively.

When the tumors reached a mean volume of 150 mm^3^, 10 animals were treated with 4HPR-HSA and 10 were the untreated control group (PBS), given slowly through the tail vein in a volume of 200 μl. The drug was administered at the dose of 1 mg/Kg every 3 days for a total of 12 administrations by tail vein injection. The experiment was terminated 48 days after the start of the treatment. At day 48, 24 h after the last administration, the mice were sacrificed and the tumors collected to determine their fenretinide content by HPLC. Immediately after removal, the tumors were homogenized by a tissue homogenizer with a 1:3 w:v ratio of physiologic saline (0.9% NaCl, w/v). They were subsequently extracted with ice-cold acetonitrile (1:1 v:v), mixed by vortex and placed in an ultrasonic bath for 10 min. The mixture obtained was then centrifuged at 10,000 × g at 4°C for 5 min and the supernatant injected directly into the HPLC system. The HPLC analysis was carried out by a Waters 2690 Separation Module equipped with a C18 (5-μm) reverse-phase column (150 × 4.6 mm) and a C18 precolumn (Perkin-Elmer, Milan, Italy). The mobile phase consisted of CH3CN : H2O : CH3COOH (75 : 23 : 2, vol/vol/vol) delivered at a flow rate of 2 ml/min. Detection was carried out with a Waters 2487 UV absorbance detector set at 340 nm [55].

We did not treat the animals with pure 4-HPR as its water insolubility would require a previous dissolution in ethanol or other water-mixable organic solvents followed by dilution with an aqueous phase before injection. As it is well known the dilution triggers drug precipitation due to the mixing of the organic solvent with water. Even if this procedure is widely used for the *in vitro* studies of poorly soluble drugs it is not a suitable experimental model in *in vivo* settings involving intravenous administrations because precipitatation of drug particles other than providing uneven bioavailability can also randomly embolize the blood vessels mainly after repeated administrations as would be needed in the present study.

### Hematoxylin and eosin (H&E) staining

Tumor samples were subjected for routine histopathological examination by standard H&E staining. Small pieces were collected in 4% paraformaldehyde for proper fixation and then were processed and embedded in paraffin wax. Sections were cut and stained with hematoxylin and eosin. Samples were observed under light microscope using an Eclipse E800 Nikon (Nikon, Tokyo, Japan). Representative images were shown. Quantitative analysis of eosin stained areas in treated tumors expressed as relative amount compared to eosin necrotic area of untreated tumors (± SD), were assessed by area counting of three fields for each of five slides per each sample at ×10 magnification by Image–ProPlus software (Immagini e Computer, Milan, Italy).

### Tunel Assay

The apoptotic cell death was assayed by *in situ* detection of DNA fragmentation using the terminal deoxynucleotidyl-transferase (TUNEL) assay. Paraffin lung cancer tissue sections (5 μm) were warmed 30 min. (64°C), deparaffinized and rehydrated. Terminal transferase mediated dUTP nick end-labeling of nuclei was performed by using APO-BrdU TUNEL Assay kit (A-23210; Molecular Probes, Eugene, OR) following the manufacturer’s protocol. Samples were observed under fluorescence microscopy using an Eclipse E800 Nikon (Nikon, Tokyo, Japan). Representative images were shown. Quantitative analysis of TUNEL positive areas expressed as relative amount of treated area compared to untreated ones, were assessed by area counting of three fields for each of five slides per each sample at ×60 magnification by Image–ProPlus software (Immagini e Computer, Milan, Italy).

### Immunohistochemistry

The sections were deparaffinised and rehydrated using 3 sequential changes of 100% xylene, 100%, 95%, 80% and 50% ethanol respectively. Briefly following removal of the paraffin samples were boiled in citric acid for 20 min. for antigen unmasking. In all cases slides were cooled with running tap water and after draining the array sections were equilibrated for 20 min. at room temperature (RT). The endogenous peroxidase activity within the rehydrated tissue was quenched using 3% hydrogen peroxide for 15 min. at RT followed by briefly washes in water and subsequently in PBS. After one hour of incubation in 2.5% of Bovine Serum Albumine/1% no fat dry milk in PBS (blocking solution), the sections were covered with primary Cav-1 (Cell Signaling Technology, Inc. Danvers) or ACSVL3 (Novus Biologicals, Cambridge) antibodies at a dilution of 1:100 and incubated for 16 hrs at 4°C. Sections were washed in PBS and the antibody signals were detected by Histofine Immunoistochemical staining kit (Nichirei Biosciences INC,Tokyo, Japan) following the manufacturer’s protocol. The sections were counterstained with haematoxylin and observed under light microscope using an Eclipse E800 Nikon (Nikon, Tokyo, Japan).

Quantitative analysis of antibody stained areas expressed as relative amount of treated samples were assessed by area counting of three fields for each of five slides per each sample at 60X magnification by Image–ProPlus software (Immagini e Computer, Milan, Italy), which allows to select and measure the antibody stained area.

### Quantitative Real Time Polymerase Chain Reaction (qRT-PCR)

Total RNA was extracted by RecoverAll Total Nucleid Acid Isolation Kit (Ambion Life Technologies, Monza, Italy) quantified using a NanoDrop® ND-1000 UV-Vis Spectrophotometer (Thermo Scientific, Wilmington, DE, USA) and cDNA was transcribed with reverse transcriptase SUPIII (Invitrogen, Carlsbad, CA, USA). The expression of mRNA was analyzed by quantitative Real Time PCR using 7500 Real Time PCR (Applied Biosystem, Life Technologies, Monza, Italy). For the analysis the following TaqMan assays (Applied Biosystems, Life Technologies, Monza, Italy) were used: Caveolin-1 (Hs00971716_m1) and ACSVL3 (HS00950760_g1). The relative gene expressions were normalized to glyceraldehyde 3-phosphate dehydrogenase (GAPDH Hs99999905_m1) and the data were presented as the fold change using the formula 2^-ΔΔCT^ as recommended by the manufacturer (User Bulletin No.2 P/N 4303859, Applied Biosystems). Data showed the average of triplicates ± SD and were representative from three independent experiments.

### Statistical Analysis

Statistical analysis was carried out using GRAPH PAD PRISM 5.0 software (San Diego, CA, USA) by applying the Student *t* test for 2 group comparisons. The differences were considered significant at p < 0.05.
